# ASSOCIATION BETWEEN WALKING ENERGETICS AND FRAGMENTED PHYSICAL ACTIVITY IN MID-TO-LATE LIFE

**DOI:** 10.1093/geroni/igz038.3175

**Published:** 2019-11-08

**Authors:** Fangyu Liu, Amal A Wanigatunga, Pei-Lun Kuo, Vadim Zipunnikov, Eleanor M Simonsick, Jennifer A Schrack

**Affiliations:** 1 Johns Hopkins Bloomberg School of Public Health, Baltimore, Maryland, United States; 2 Johns Hopkins University Bloomberg School of Public Health, Baltimore, Maryland, United States; 3 Longitudinal Studies Section, Intramural Research Program, National Institute on Aging, Baltimore, Maryland, United States; 4 Johns Hopkins University, Baltimore, Maryland, United States

## Abstract

Physical activity becomes increasingly fragmented with age, and may be an early marker of functional decline. Energy regulation has been linked with functional decline, yet whether the energy needed for walking, a common type of physical activity, is related to fragmentation of physical activity remains unknown. The study population included 493 participants aged 50-93 years from the Baltimore Longitudinal Study of Aging. Energetic measures included the energetic cost of usual-paced overground walking (ml/kg/m), the average energy expended (ml/kg/min) during a rapid-paced 400-m walk, and a cost-to-capacity ratio between the energy expended during 5-min treadmill walk (0.67 m/s, 0% grade) and the energy expended during the 400-m walk. Activity fragmentation was extracted from accelerometer data collected over ≥3 valid days and quantified via an active-to-sedentary transition probability (ASTP). Associations between the energetic measures and ASTP were assessed using multivariate linear regression models. Interactions between energetics and total daily physical activity, quantified as total log-transformed activity counts (TLAC), were also assessed. After adjusting for TLAC, demographics, body composition and comorbidity, higher cost-to-capacity ratio was associated with 3.51% greater fragmented physical activity (p=0.005). Energetics by TLAC interactions revealed that lower rapid-paced walking energy expenditure and higher cost-to-capacity ratio were only significantly associated with greater fragmentation in the most sedentary participants (p<0.01 for both). Our results suggest that deterioration of walking efficiency may manifest as a more fragmented physical activity profile, especially among sedentary adults. Future longitudinal studies to understand whether declining walking efficiency predicts the onset and progression of activity fragmentation are warranted.

